# Milwaukee Shoulder Syndrome

**DOI:** 10.1155/2014/458708

**Published:** 2014-01-15

**Authors:** Channa Vasanth Nadarajah, Immo Weichert

**Affiliations:** Department of Acute Medicine, The Ipswich Hospital NHS Trust, Ipswich, Suffolk IP4 5PD, UK

## Abstract

Milwaukee shoulder syndrome (MSS) is a rare destructive, calcium phosphate crystalline arthropathy. It encompasses an effusion that is noninflammatory with numerous aggregates of calcium hydroxyapatite crystals in the synovial fluid, associated with rotator cuff defects. We describe a patient that presented with recurrent shoulder pain and swelling with characteristic radiographic changes and MSS was confirmed on aspiration of the synovial fluid.

## 1. Case

An 89-year-old lady presented to the medical assessment unit with a four-day history of melaena associated with shortness of breath on exertion, postural dizziness, and lethargy. This was secondary to esophagitis and gastric erosions. During her hospital stay she developed a rapid onset, painful swelling of her right shoulder with a limited range of movement. There was no history of recent trauma to the right shoulder. This was her third presentation; previous ones had been followed up by her general practitioner.

Past medical history included well-controlled asthma, hypertension, and atrial fibrillation. There was no history of diabetes, hyperparathyroidism, or syphilis. She was an ex-smoker with a 10-pack-year history and consumed minimal amounts of alcohol only.

**Figure 1:**
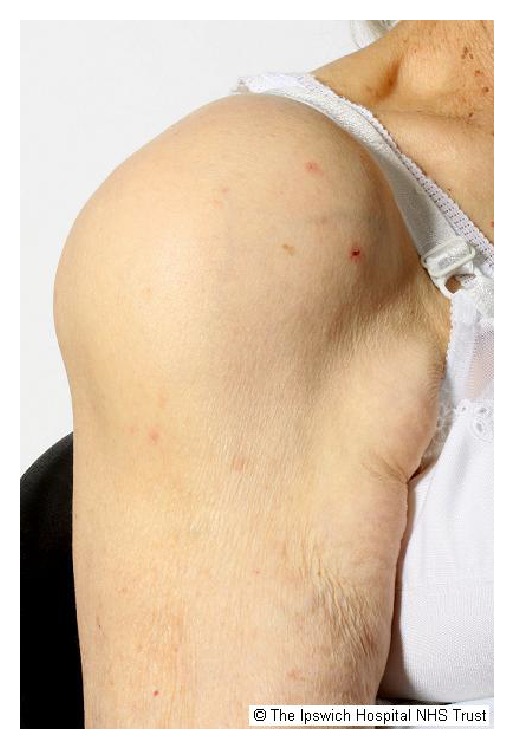
Submission remains copyrighted by the Ipswich Hospital NHS trust as per trust policy—we have permission from the patient and also from the trust to publish.

On examination, she was pale, with no rashes or bruises. There was a large right shoulder effusion. Both active and passive movements were limited.  Figure 1 shows the patient's large right shoulder swelling which was warm to touch and tender with no erythema.

**Figure 2:**
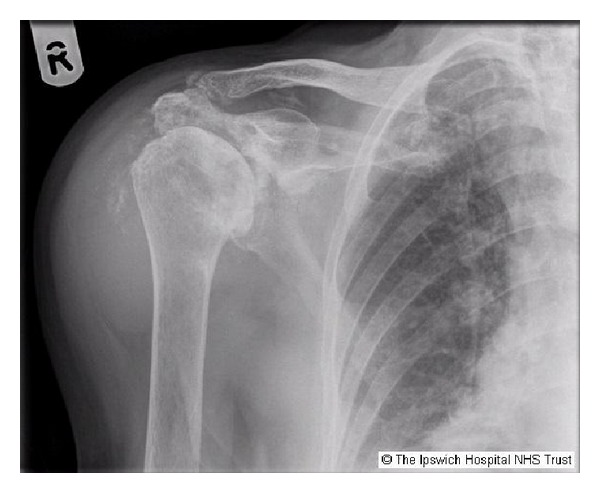
Submission remains copyrighted by the Ipswich Hospital NHS trust as per trust policy—we have permission from the patient and also from the trust to publish.

Plain radiograph of the affected shoulder showed joint space narrowing, subchondral sclerosis, destruction of subchondral bone, soft-tissue swelling, capsular calcifications, and intra-articular loose bodies (Figure 2).

Arthrocentesis was performed and 250 mLs of haemorrhagic fluid was aspirated. Analysis of the synovial fluid showed a noninflammatory cell count with leukocytes 721/mm^3^ which were predominantly neutrophils. Gram stain was negative; no organisms were cultured and cytology analysis was negative, as was VDRL on the synovial fluid. The aspirate stained bright orange-red with alizarin red S, indicating the presence of calcium hydroxyapatite crystals. The diagnosis of Milwaukee shoulder syndrome was made and she was treated conservatively with colchicine and physiotherapy with a good outcome.

## 2. Discussion

The term Milwaukee shoulder syndrome (MSS) was first used in 1981 to describe four elderly women in Milwaukee in the state of Wisconsin, USA, with recurrent bilateral shoulder effusions, radiographic evidence of severe destructive changes of the glenohumeral joints, and massive tears of the rotator cuff [1–3]. The term rapid destructive arthritis of the shoulder was introduced in 1982 to describe six elderly females with spontaneous large glenohumeral effusions, mild pain, and tears of the rotator cuff [4]. Apatite-associated destructive arthritis and idiopathic destructive arthritis were introduced to illustrate rotator cuff tear arthropathy of the shoulder in 1983 [5].

MSS is a destructive, calcium phosphate crystalline arthropathy; it encompasses an effusion that is noninflammatory with numerous aggregates of calcium hydroxyapatite crystals in the synovial fluid, associated with rotator cuff defects [6, 7]. Calcium hydroxyapatite crystal disease is characterised by recurrent painful periarticular calcific deposits in tendons, soft tissues, or intra-articular surfaces [6]. The shoulder is most frequently involved; however, wrists, hands, elbows, neck, lumbar spine, hips, knees, and feet may also be affected [6].

The pathophysiology is believed to be the intra-articular calcium hydroxyapatite deposition. This induces the release of lysosomal enzymes, which attack the periarticular tissues, including the rotator cuff. The presence of activated collagenase and neutral proteases capable of disrupting articular cartilage and enzymes has been demonstrated in cultured synovial lining cells [3].

MSS occurs in elderly patients typically aged 60–90 years [3, 6, 8]. There is a female preponderance in the ratio of 4 : 1 [6–8]; the higher life expectancy of women may be contributory. Unilateral shoulder joint involvement is more common and seen in the dominant side; however, in case of bilateral shoulder disease this is almost always more advanced on the dominant side. Risk factors for MSS are listed in Table 1.

Radiographic changes on plain X-ray show joint space narrowing, subchondral sclerosis with cyst formation, destruction of subchondral bone, soft-tissue swelling, capsular calcifications, and intra-articular loose bodies. Ultrasonography of the shoulder usually shows fluid collection and marked synovial proliferation. Magnetic resonance imaging demonstrates a large effusion, rotator cuff tears, narrowing of the glenohumeral joint, thinning of the cartilage, and destruction of subchondral bone [7–9].

**Figure 3:**
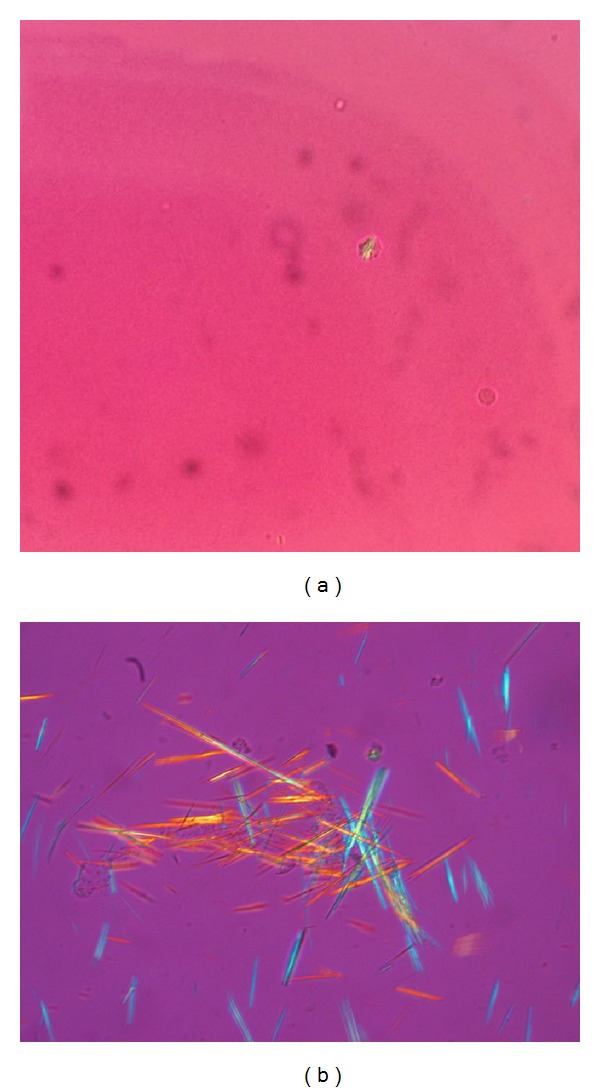
(From Wikipedia) Licensed under the Creative Commons Attribution-Share Alike 3.0 Unported license: (a) [12], (b) [13].

Unlike monosodium urate (MSU) and calcium pyrophosphate dehydrate crystals (CPPD), identifying calcium hydroxyapatite crystals has been elusive for many years, as they do not appear under plain and polarized microscopy [10, 11].  Figure 3 shows (a) CPPD and (b) MSU identified under polarized light microscopy of synovial fluid [12, 13].

However, globular clumps of hydroxyapatite crystals may appear using plain light microscopy but are not birefringent. Such clumps have been described as “shiny coins,” although they often lack distinctive qualities or are often not observed [10]. The introduction of alizarin red S stain has allowed for a simple, rapid method to identify clumps of calcium hydroxyapatite crystals that produce a characteristic “halo” of orange-red stain [10, 11, 14].

Aspirated synovial fluid is typically haemorrhagic, noninflammatory, and positive for calcium apatite crystals. Leukocyte count is typically low, usually less than 800/mm^3^. Damage to the rotator cuff precedes crystal deposition and shedding of crystals is responsible for sudden, acute episodes of inflammation. Occasionally synovial fluid may reveal calcium hydroxyapatite and CPPD crystals, a condition termed mixed calcium phosphate deposition disease [6]. It is commonly seen if radiographs show extensive cartilage calcification and diffuse intra-articular and capsular calcification or dense, homogeneous calcific collection of the tendons. Histopathological studies of the synovium have shown synovial lining cell hyperplasia, giant cell formation, and deposition of fibrin and calcium phosphate crystals. These findings help differentiate between other crystal deposition arthropathies.  Table 2 lists the most important differential diagnoses for MSS.

Treatment is usually supportive; resting the affected joint and the use of nonsteroidal anti-inflammatory agents have shown to be very effective. Colchicine has been shown to be effective in the management of MSS [15]. Physiotherapy has a major role in the management of the condition. It provides the required exercise to maintain the range of motion and strengthen the surrounding muscles. For large effusions arthrocentesis is beneficial. Surgical intervention, such as partial or complete arthroplasty, is considered in severe or advanced degenerative changes, provided there are no contraindications.

This case was a rare cause of an acutely swollen joint. The patient presented with recurrent shoulder pain and swelling with characteristic radiographic changes and MSS was confirmed on aspiration of the synovial fluid. She responded well to conservative management. This case highlights the importance of considering calcium hydroxyapatite crystal arthropathy as a potential cause for sudden onset monoarthritis.

Detection and identification of crystals in synovial fluid are critical for any diagnosis and treatment of crystalline arthropathy. In appropriate clinical settings using the simple preparation of alizarin red S stain can help confirm MSS.

## Figures and Tables

**Table 1:** Associated risk factors for Milwaukee shoulder syndrome.

(1) Trauma or overuse	
(2) Calcium pyrophosphate dehydrate crystal deposition	
(3) Neuroarthropathy	
(4) Dialysis arthropathy	
(5) Denervation	
(6) Female gender	
(7) Advanced age	

**Table 2:** Differential diagnosis of Milwaukee shoulder syndrome.

(1) Rapidly destructive or progressive arthropathy	
(2) Septic arthritis	
(3) Neuropathic arthropathy	
(4) Osteonecrosis	
(5) Inflammatory arthritis	
(6) Crystal-associated arthropathy	
(7) Arthropathy of late syphilis	

## Learning Point

The learning point is to increase awareness of a rare cause of an acutely swollen joint.

MSS is a destructive, calcium phosphate crystalline arthropathy; it encompasses an effusion that is noninflammatory with numerous aggregates of calcium hydroxyapatite crystals in the synovial fluid, associated with rotator cuff defects.
